# Lung Function in a Multiethnic U.S. Cohort of Adolescents and Adults Born Preterm in the New BPD Era

**DOI:** 10.1002/ppul.71226

**Published:** 2025-08-08

**Authors:** Nataly J. Sanchez‐Solano, Tyler J. Wall, Gregory P. Barton, Dan M. Dane, Connie C. W. Hsia, Kara N. Goss

**Affiliations:** ^1^ Department of Pediatrics University of Texas Southwestern Medical Center Texas USA; ^2^ Department of Internal Medicine University of Texas Southwestern Medical Center Texas USA; ^3^ Parkland Health University of Texas Southwestern Medical Center Texas USA

**Keywords:** bronchopulmonary dysplasia, obstructive lung disease, premature birth, respiratory function tests, socioeconomic disparities in health

## Abstract

**Rationale:**

Extreme preterm birth is a recognized risk factor for reduced pulmonary function over the lifespan. However, data are lacking in U.S.‐born adults and in multiethnic populations.

**Objective:**

To comprehensively evaluate lung function in adolescents and young adults born premature, and to assess for potential impact of neonatal, racial/ethnic, and socioeconomic risk factors on long‐term lung function.

**Methods:**

Preterm participants aged 12–40 years of age were recruited from the Parkland Hospital Neonatal ICU Registry (Dallas County, Texas). Preterm and term‐born participants completed pulmonary function testing including spirometry, lung volumes, diffusion capacity, and respiratory muscle forces, using Global Lung Initiative race‐neutral reference equations. Influence of racial/ethnic background, neonatal factors, and socioeconomic ratings were assessed for preterm participants.

**Results:**

Preterm participants (*n* = 105; gestational age 29.5 ± 2.5 weeks; current age 26 ± 6 yrs) were significantly more likely than term participants (*n* = 48; gestational age 39.2 ± 1.1 weeks; current age 29 ± 7 yrs) to have an obstructive or restrictive ventilatory defect (34.4% vs 14.6%, OR 3.06, 95% CI 1.26–7.93), reduced diffusion capacity (16.4% vs 2.1%, OR 9.18, 95% CI 1.47–98.3), and impaired respiratory muscle strength (27.7% vs 4.4%, OR 8.22, 95% CI 2.10–36.2). Neonatal risk factors included history of bronchopulmonary dysplasia and lower weight for gestational age percentile. Lung function was worse in preterm‐born Non‐Hispanic Black participants compared to preterm‐born Hispanic White participants, which could not be explained by available neonatal or socioeconomic factors.

**Conclusion:**

While most moderately to extremely preterm‐born individuals have normal lung function, obstructive and restrictive ventilatory defects, reduced diffusion capacity, and impaired respiratory muscle strength are common.

## 1 Introduction

Preterm birth, defined as birth before 37 weeks gestation, affects roughly 1 in 10 live births in the United States. Critically, fetal lung development is incomplete at earlier gestations, and preterm birth may impair subsequent lung development and growth, increasing risk for life‐long lung disease. Pulmonary complications into early adulthood for individuals with a history of prematurity have been described, such as early onset chronic obstructive pulmonary disease (COPD), reactive airway disease, reduced exercise capacity, and pulmonary hypertension [1, 2, 3]. Abnormal lung growth leading to dysanapsis has also been reported [4]. Extremes of prematurity and neonatal diagnosis of bronchopulmonary dysplasia (BPD) are among several risk factors for long‐term disease [5]. The vast majority of preterm‐born adults studied to date come from primarily Caucasian cohorts, with most born internationally in the 1970s or 1980s [1, 6], and thus may not reflect the current U.S. population. Furthermore, the impact of race, ethnicity, and socioeconomic status remain unclear.

There is a critical unmet need to understand the implications of moderate to extreme preterm birth on long‐term lung function in a multi‐ethnic population born in the modern‐day post‐surfactant era. Here, we utilized a unique U.S.‐born multi‐ethnic preterm cohort born and cared for in the Parkland Hospital Neonatal Intensive Care Unit (NICU), the safety net hospital of Dallas County, Texas, to comprehensively study pulmonary function in adolescents and young adults born ≤ 32 weeks gestation or < 1500 gram birth weight. The primary objective of this study was to evaluate whether adolescents and adults with a history of moderate to extreme prematurity have evidence of reduced lung function, as documented by pulmonary function testing (PFT) including spirometry, lung volumes, diffusion capacity, and respiratory muscle forces. Given the lack of prior assessments in racial minority groups, assessment of whether lung function after preterm birth differed by racial/ethnic status was a secondary objective. The impact of neonatal and socioeconomic risk factors for long‐term reduced lung function were also assessed.

## 2 Methods

### 2.1 Participants

The Cardiopulmonary Sequelae in Adolescents and Adults Born Preterm Study was a long‐term follow up study from the Parkland Hospital Neonatal Intensive Care Unit (NICU) Registry [7, 8]. This single‐center cross‐sectional study prospectively enrolled term and preterm adolescents and young adults aged 12–40 from June 2021 to September of 2023. Preterm participants were identified as eligible if born at ≤ 32 weeks gestation or with a birthweight of < 1500 grams at Parkland Hospital. Term participants were eligible if they were born ≥ 37 weeks and had birthweight of > 2500 g, with birth history self‐reported. Term participants were excluded if they had a body mass index (BMI) > 40 kg/m^2^, pre‐existing cardiopulmonary disease, or use of cardiac or pulmonary medications. The complete study included health questionnaires, focused cardiopulmonary exam, echocardiography [8], comprehensive PFT, and overnight oximetry [7]; here we present results related to PFT. The study protocol was reviewed and approved by the UT Southwestern Institutional Review Board (STU 2020‐1332). All participants provided written consent, with assent obtained for adolescent participants.

### 2.2 Pulmonary Function Testing

Participants completed the St. George Respiratory Questionnaire (SGRQ) to assess for respiratory symptomatology [9]. All participants completed the following comprehensive PFT in accordance with ATS/ERS guidelines [10, 11, 12] [1]: *spirometry* (FEV1 = forced expiratory volume in 1 s; FVC = forced vital capacity; FEF25‐75 = forced mid‐expiratory flow) [2]; *lung volumes* (TLC = total lung capacity; VC = vital capacity; IC = inspiratory capacity; FRC = functional residual capacity; ERV = expiratory reserve volume; RV = residual volume) [3]; *lung diffusion capacity* (DLCO = diffusion capacity for carbon monoxide; DLCO Adj = diffusion capacity for carbon monoxide adjusted for hemoglobin; VA = alveolar volume); and [4] *respiratory forces* (MIP = maximal inspiratory pressure; MEP = maximal expiratory pressure; MVV = maximal voluntary ventilation). Predicted values were calculated using Global Lung Initiative (GLI) datasets [13]. GLI race neutral predicted and Z‐score values are presented, with the exception of FEF25‐75 values for which GLI race neutral values were unavailable and GLI race‐specific datasets were used. For disease classification, the ERS/ATS technical standard on interpretation was applied using Z‐score defined upper and lower limits to define pathology following interpretation algorithms [12].

Hispanic White and Non‐Hispanic Black were the two primary ethnicity/race combinations in the preterm population, accounting for 91% of enrolled preterm participants, and thus, preterm subgroup analysis was completed. To probe the contribution of socioeconomic factors, birth and adult addresses were geocoded for preterm participants and matched by census tract information to the Childhood Opportunity Index (COI) 2.0 2015 data set [14, 15]. Nationally standardized Z‐scores for subdomains of education, health/environment, social/economic, and total COI scores were computed.

### 2.3 Statistical Analysis

Statistical analysis was performed using Graphpad Prism version 10. Univariable comparisons of baseline/demographic characteristics, pulmonary function, and respiratory symptoms between term and preterm groups as well as preterm Non‐Hispanic Black and Hispanic groups were conducted using Chi‐square test for categorical variables, and unpaired *t*‐test (or Wilcoxon rank sum test if necessary) for continuous variables. Odds ratios were computed using Baptist‐Pike method for various pulmonary function profiles. Least squares multivariable regression analyses were conducted to assess for the impact of neonatal and socioeconomic factors between the preterm groups. Multicollinearity was assessed using the Variance Inflation Factor (VIF) with all values being less than < 2.5.

*Additional methodologic details are provided in the online supplement*.

## 3 Results

### 3.1 Baseline Characteristics

A total of 153 subjects were included in this study, of whom 105 were preterm (average gestational age 29.5 ± 2.5 weeks) and 48 were term (average gestational age 39 ± 1.1 weeks). Two preterm participants from the original data set were excluded due to inability to reliably complete any PFT. Baseline characteristics, including demographics, anthropometrics, and summary level neonatal characteristics are listed in Table 1. Preterm participants were slightly younger and more likely to be of Black race or Hispanic ethnicity, were overall shorter, and had significantly higher BMI compared to the term participants. Preterm participants were also more likely to self‐report a history of asthma, had similar tobacco use history, and had reduced strength as determined by lower handgrip muscle forces. Preterm Hispanic patients were younger, born in more recent birth years, and at earlier gestational age compared to Preterm Black participants, consistent with the changing demographics and neonatal survival of the Parkland NICU population [16]. To understand the expected generalizability of the results, the enrolled preterm participants were compared to the eligible preterm participants from the Parkland NICU Registry (Table 2). Enrolled preterm participants were slightly lower gestational age and birth weight than the overall eligible registry population, with higher rates of cardiopulmonary morbidity and longer length of hospital stay, suggesting a slightly sicker enrolled preterm population. Rates of neonatal neurologic and infectious complications were similar.

**Table 1:** Baseline characteristics.

Enrollment characteristics	Term (*n* = 48)	Preterm (*n* = 105)	*p*‐value*	Preterm Non‐Hispanic Black (*n* = 27)	Preterm Hispanic (*n* = 69)	*p*‐value**
Age, mean (range)	29 (13–40)	26 (16‐36)	**0.02**	30 (21–34)	24 (16–35)	**< 0.001**
Birth year, mean (range)	1994 (‘82‐’09)	1996 (‘86‐‘06)	**0.04**	1991 (‘87‐‘01)	1998 (‘86‐‘06)	**< 0.001**
Female, *n* (%)	29 (60.4)	70 (66.7)	0.47	22 (78.6)	45 (64.3)	0.23
Hispanic/Latinx ethnicity, *n* (%)	16 (33.3)	70 (66.7)	**< 0.01**	0 (0)	69 (100)	**< 0.001**
Race, *n* (%)			**< 0.01**			**< 0.001**
Black or African American	5 (10.4)	28 (26.7)	—	27 (100)	0 (0)	—
White	30 (62.5)	73 (69.5)	—	—	—	—
Asian	9 (18.8)	3 (2.9)	—	—	—	—
Other	4 (8.3)	1 (1.0)	—	—	—	—
Height, mean (SD), cm	170 (10)	162 (10)	**< 0.01**	168 (11)	160 (9)	**< 0.001**
Weight, mean (SD), kg	74.3 (16.3)	79.2 (23.4)	0.14	92.4 (29.8)	74.1 (19.2)	**< 0.001**
BMI, mean (SD), kg/m2	25.7 (4.5)	29.9 (7.4)	**< 0.01**	32.7 (9.4)	28.9 (6.5)	**0.03**
Current or former smoker, *n* (%)	10 (20.8)	35 (33.3)	0.13	13 (41.9)	18 (25.7)	0.05
Self‐reported asthma diagnosis, *n* (%)	4 (8.3)	29 (27.6)	**< 0.01**	9 (32.1)	16 (22.9)	0.44
Handgrip strength, mean (SD), kg	37.2 (12.9)	32.9 (11.2)	**0.04**	34.9 (12.3)	30.8 (10.0)	0.10
**Neonatal characteristics**						
Birth weight, mean (SD), g	3332 (468)	1311 (396)	**< 0.01**	1351 (389.1)	1277 (391.3)	0.40
Gestational age (GA), mean (SD), weeks	39.2 (1.1)	29.5 (2.5)	**< 0.01**	30.4 (1.1)	29.1 (2.5)	**0.02**
Small for gestational age, *n* (%)	5 (11)	33 (31)	**0.01**	8 (29.6)	24 (34.8)	0.81
Weight for GA Z‐score (SD)	−0.02 (1.14)	−0.85 (1.65)	**< 0.01**	−1.50 (1.7)	−0.66 (1.6)	**0.02**
Weight for GA %ile (SD)	49.3 (30.0)	34.6 (32.6)	**< 0.01**	21.0 (22.6)	38.3 (34.8)	**0.02**

*Note:* Data are presented as *n* (%) or mean (SD) unless otherwise specified. Bold values are statistically significant at *p* < 0.05.

Abbreviations: BMI = body mass index, GA = gestational age, SD = standard deviation.

* *p*‐value calculated comparing term vs preterm or

** *p*‐value calculated comparing preterm Non‐Hispanic Black versus preterm Hispanic with Wilcoxon rank sum test for non‐normally distributed continuous variables, *T*‐test for normally distributed continuous variables, and chi‐squared for categorical variables.

**Table 2:** Neonatal characteristics comparing registry database with enrolled preterm subjects.

Characteristic	Total (*N* = 6000)	Registry (*N* = 5895)	Enrolled (*N* = 105)	*p* value
**Demographics**				
Birth weight, mean (SD), g	1474 (448)	1477 (448)	1311 (396)	**< 0.01**
Birth weight classification, *n* (%)				**0.03**
ELBW (< 1000 g)	922 (15)	897 (15)	25 (24)	
VLBW (1000–1499 g)	2335 (39)	2290 (39)	45 (43)	
LBW (1500–2499 g)	2664 (44)	2629 (45)	35 (33)	
NBW (≥ 2500 g)	79 (1)	79 (1)	0	
GA, mean (SD), weeks	31 (4)	31 (4)	30 (3)	**< 0.01**
GA classification, *n* (%)				**< 0.01**
Extremely preterm (< 28 weeks)	989 (16)	958 (16)	31 (30)	
Very preterm (28 to < 32 weeks)	3054 (51)	3007 (51)	47 (45)	
Moderate preterm (32–34 weeks)	1681 (28)	1658 (28)	23 (22)	
Late preterm (35–36 weeks)	193 (3)	189 (3)	4 (4)	
Term (≥ 37 weeks)	83 (1)	83 (1)	0	
Total length of stay, median (IQR), days	33 (38)	33 (38)	47 (52)	**< 0.01**
**Neonatal diagnoses**				
*Respiratory*				
Invasive ventilation, median (IQR), days	0 (2)	0 (2)	1 (5)	**< 0.01**
Noninvasive ventilation, median (IQR), days	0 (3)	0 (3)	1 (12)	**< 0.01**
Total ventilation, median (IQR), days	1(6)	1 (6)	3 (20)	**< 0.01**
Duration of oxygen therapy, median (IQR), days	5 (36)	5 (36)	3.5 (43)	0.28
Bronchopulmonary dysplasia, *n* (%)	524 (9)	508 (9)	16 (15)	**0.02**
Antenatal steroids, *n* (%)	1351 (41)	1317 (41)	34 (54)	**0.04**
Surfactant, *n* (%)	1350 (35)	1320 (35)	30 (43)	0.15
Chronic lung disease by CXR, *n* (%)	894 (15)	865 (15)	29 (28)	**< 0.01**
Apnea of prematurity, *n* (%)	3205 (53)	3136 (53)	69 (66)	**0.01**
*Cardiovascular*				
Patent ductus arteriosus, *n* (%)	981 (16)	958 (16)	23 (22)	0.12
Congenital heart disease, *n* (%)	103 (2)	95 (2)	8 (8)	**< 0.01**
*Neurological*				
Intraventricular hemorrhage, *n* (%)				
No intraventricular hemorrhage	4737 (79)	4657 (79)	80 (76)	0.48
Grade I–II	977 (16)	959 (16)	18 (17)	0.81
Grade III–IV	286 (5)	279 (5)	7 (7)	0.36
*Infectious*				
Sepsis, *n* (%)	1952 (33)	1910 (33)	42 (40)	0.13
Necrotizing enterocolitis, *n* (%)	415 (7)	404 (7)	11 (10)	0.15

*Note:* Data are presented as *n* (%) or mean (SD) unless otherwise specified. *p*‐value calculated comparing preterm vs.versus term with Wilcoxon rank sum test for non‐normally distributed continuous variables, *T*‐test for normally distributed continuous variables, and chi‐squared for categorical variables. Missing values from antenatal steroids (Total = 2734, Registry = 2712, Enrolled = 42), surfactant (Total = 2123, Registry = 2088, Enrolled = 35). Enrolled percentages are calculated for preterm group only and are based on *N* = 105. Bold values are statistically significant at *p* < 0.05.

Abbreviations: CXR = chest xray, ELBW = extremely low birth weight, GA = gestational age, IQR = interquartile ranges, LBW = low birth weight, NBW = normal birth weight, SD = standard deviation, VLBW = very low birth weight.

### 3.2 Pulmonary Function Tests and Respiratory Symptoms

**Figure 1:**
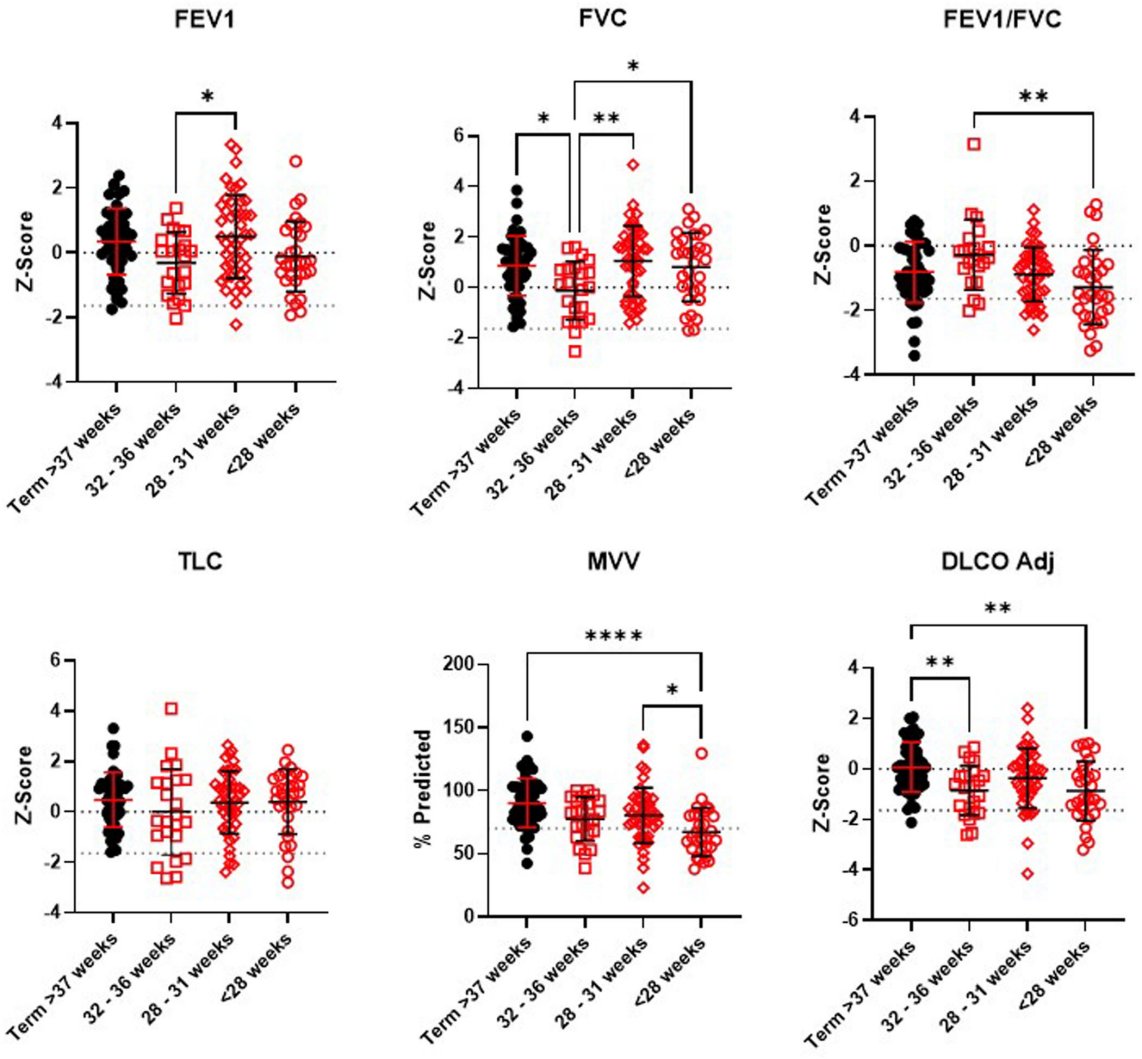
Key pulmonary function parameters by gestational age categories. Error bars represent mean with standard deviation. FEV1 = forced expiratory volume in 1 s, FVC = forced vital capacity, TLC = total lung capacity, MVV = maximal voluntary ventilation, DLCO Adj = diffusion capacity for carbon monoxide adjusted for hemoglobin. [Color figure can be viewed at wileyonlinelibrary.com]

Comprehensive lung function was compared between term and preterm participants. There were no statistically significant differences in FEV1, FVC, FEV1/FVC ratio, or lung volume %‐predicted or Z‐scores between term and preterm participants (Table 3), with the exception of ERV. However, diffusion capacity percent predicted and Z‐scores were significantly reduced in the preterm group compared with term subjects. Additionally, all measures of muscle forces including MVV, MIP, and MEP were significantly lower in adolescents and adults born preterm. Reductions in MIP, and to a lesser extent MEP, were strongly associated with peripheral muscle strength as measured by handgrip in both term and preterm participants (MIP term R^2^: 0.36, *p* < 0.0001; preterm R^2^: 0.29, *p* < 0.0001; Figure E1). Preterm participants also reported significantly greater respiratory symptoms across all domains of the St. George Respiratory Questionnaire (SGRQ). Key pulmonary function parameters are also presented graphically by gestational age in Figure 1.

**Table 3:** Pulmonary function and respiratory symptoms in term and preterm participants.

Spirometry	Term (*n* = 48)	Preterm (*n* = 105)	*p*‐value
FEV1, *Z*‐Score	0.35 (1.02)	0.14 (1.20)	0.30
FEV1, % Predicted	104.5 (13.8)	101.5 (16.0)	0.27
FVC, *Z*‐Score	0.86 (1.18)	0.73 (1.42)	0.61
FVC, % Predicted	112.0 (16.5)	110.2 (19.7)	0.58
FEV1/FVC, Z‐score	−0.81 (0.94)	−0.90 (1.05)	0.63
FEV1/FVC ratio	79.7 (7.5)	80.4 (6.82)	0.53
FEF25‐75, *Z*‐score	−0.37 (0.83)	−0.53 (0.84)	0.27
FEF25‐75, % Predicted	92.0 (19.5)	88.5 (19.7)	0.30
**Lung volumes**			
TLC, *Z*‐score	0.48 (1.08)	0.30 (1.38)	0.43
TLC, % Predicted	106.1 (13.2)	104.4 (16.9)	0.53
VC, *Z*‐score	0.63 (1.32)	0.35 (1.61)	0.31
VC, % Predicted	107.5 (15.8)	104.1 (19.1)	0.28
IC, *Z*‐score	0.52 (1.41)	0.82 (1.55)	0.26
IC, % Predicted	108.9 (24.1)	114.2 (26.9)	0.24
FRC, *Z*‐score	0.13 (1.12)	−0.26 (1.31)	0.08
FRC, % Predicted	104.6 (23.2)	97.4 (26.9)	0.11
ERV, *Z*‐score	0.37 (1.27)	−0.21 (1.49)	**0.02**
ERV, % Predicted	116.2 (43.9)	97.5 (44.9)	**0.02**
RV, *Z*‐score	0.05 (0.76)	0.07 (1.34)	0.92
RV, % Predicted	103.5 (28.0)	108.0 (51.8)	0.58
RV/TLC, *Z*‐score	−0.07 (0.82)	0.07 (1.35)	0.49
RV/TLC, % Predicted	98.3 (24.6)	102.7 (40.4)	0.48
**Diffusion capacity**			
DLCO, *Z*‐score	0.10 (1.00)	−0.54 (1.12)	**< 0.01**
DLCO, % Predicted	101.9 (14.2)	94.0 (14.3)	**< 0.01**
DLCO Adj, *Z*‐score	0.07 (0.99)	−0.65 (1.13)	**< 0.01**
DLCO Adj, % Predicted	101.5 (13.8)	92.3 (14.5)	**< 0.01**
VA, *Z*‐score	0.24 (1.34)	−0.40 (1.63)	**0.02**
VA, % Predicted	103.2 (14.8)	96.5 (17.1)	**0.02**
DLCO/VA, *Z*‐score	−0.11 (1.10)	−0.31 (1.02)	0.27
DLCO/VA, % Predicted	100.1 (15.5)	98.54 (13.7)	0.53
**Muscle forces**			
MIP, mmHg	81.3 (20.7)	71.3 (27.6)	**0.03**
MEP, mmHg	88.7 (34.9)	63.3 (28.2)	**< 0.01**
MVV, % Predicted	90.2 (19.4)	75.8 (20.5)	**< 0.01**
**St. George Respiratory Questionnaire**			
Symptom score (IQR)	4.4 (0‐10.9)	8.8 (0‐21.4)	**0.01**
Activity score (IQR)	5.9 (0‐12.7)	18.5 (0‐33.7)	**< 0.01**
Impact score (IQR)	0 (0)	3.6 (0‐12.5)	**< 0.01**
Total score (IQR)	2.11 (0‐6.1)	9.3 (3.7‐20.4)	**< 0.01**

*Note:* Data are presented as mean (SD) unless otherwise specified. St. George respiratory questionnaire (SGRQ) data are presented as median (IQR). Percent predicted and Z‐scores are calculated using Global Lung Initiative (GLI) race neutral values, with exception of FEF25‐75 values for which GLI race neutral values were unavailable and GLI race‐specific datasets were used. *p*‐value calculated comparing preterm vs.versus term with unpaired t test for normally distributed continuous variables (lung function variables) or Wilcoxon rank sum test for non‐normally distributed continuous variables (SGRQ variables). Bold values are statistically significant at *p* < 0.05.

Abbreviations: DLCO = diffusion capacity for carbon monoxide, DLCO Adj = diffusion capacity for carbon monoxide adjusted for hemoglobin, ERV = expiratory reserve volume, FEF25‐75 = forced mid‐expiratory flow, FEV1 = forced expiratory volume in 1 s, FRC = functional residual capacity, FVC = forced vital capacity, IC = inspiratory capacity, IQR = interquartile range, MEP = maximal expiratory pressure, MIP = maximal inspiratory pressure, MVV = maximal voluntary ventilation, RV = residual volume, TLC = total lung capacity, VA = alveolar volume, VC = vital capacity.

Despite the appearance of similar spirometry and lung volumes between term and preterm participants, application of the 2022 ERS interpretive strategies for PFT demonstrated multiple abnormal pulmonary function profiles (Table 4). Specifically, 34.3% of preterm‐born adolescents demonstrated an abnormal obstructive or restrictive ventilatory pattern, compared to 14.6% of term‐born controls (*p* = 0.01, OR 3.06). Rates of obstructive, dysanapsis and hyperinflation patterns were non‐significantly higher in the preterm participants, while restriction was significantly more common in the preterm participants (11.4% of the preterm population). Obstructive profiles were most prevalent in the extremely preterm group (35%), compared to the very preterm (21%) and moderately preterm (13%) groups. Notably, abnormally low DLCO was present in 16.4% of the preterm participants, versus only 2.1% of term (*p* = 0.01, OR 9.18), with a variety of impaired diffusion profiles. There was not a clear dose–response of prematurity, as 12.9%, 12.8%, and 26% had reduced DLCO (extreme, very, and moderately preterm, respectively). Neuromuscular weakness was also significantly more common in the preterm population, affecting 42% of the extremely preterm group versus 15% and 22% of the very and moderately preterm groups respectively.

**Table 4:** Pulmonary function profiles in term and preterm participants.

Pattern	Definition	Termn *N* (%)	Preterm *N* (%)	*p*‐value	OR	95% CI
Abnormal volumes	Obstructive or restrictive pattern	7 (14.6%)	36 (34.3%)	**0.01**	3.06	1.26–7.93
Obstruction	FEV1/FVC < LLN; FVC > LLN	7 (14.6%)	25 (23.8%)	0.28	1.79	0.75–4.54
‐ Dysanapsis	FEV1/FVC < LLN; FVC > ULN	4 (8.3%)	12 (11.4%)	0.77	1.42	0.48–4.20
Restriction	TLC < LLN	0 (0%)	12 (11.4%)	**0.02**	13.0	0.75–224
Hyperinflation	RV/TLC > ULN	2 (4.2%)	12 (11.4%)	0.23	2.97	0.69–13.7
Low DLCO	DLCO Adj < LLN	1 (2.1%)	17 (16.4%)	**0.01**	9.18	1.47–98.3
‐ Primary vascular abnormality or emphysema	DLCO Adj < LLN, VA Normal/High	1 (2.1%)	6 (5.8%)	0.43	2.88	0.45–33.7
‐ Low lung volume, poor expansion	DLCO Adj < LLN, VA < LLN, K_co_ > ULN	0 (0%)	2 (1.9%)	0.99	2.37	0.11–50.2
‐ Loss of capillary and lung volume	DLCO Adj < LLN, VA < LLN, K_co_ Normal/Low	0 (0%)	9 (8.7%)	0.06	9.65	0.55–169
Neuromuscular weakness	MVV < 70% pred; and MIP or MEP < 60 mmHg	2 (4.4%)	26 (27.7%)	**0.001**	8.22	2.01–36.2

*Note:* Data are presented as N (%). *p*‐value calculated for categorical variables using Fisher′s exact test. Odds ratio (OR) and confidence intervals (CI) computed using Baptista‐Pike method. Bold values are statistically significant at *p* < 0.05.

Abbreviations: DLCO Adj = diffusion capacity for carbon monoxide adjusted for hemoglobin, FEV1 = forced expiratory volume in 1 s, FVC = forced vital capacity, K_co_ = Carbon monoxide transfer coefficient, LLN = lower limit of normal, defined as < 5th percentile, MEP = maximal expiratory pressure, MIP = maximal inspiratory pressure, MVV = maximal voluntary ventilation, RV = residual volume, TLC = total lung capacity, VA = alveolar volume.

### 3.3 Impact of Race and Ethnicity on Lung Function

Prior long‐term pulmonary follow up studies have included greater than 90% Non‐Hispanic white participants. Thus, we independently evaluated the impact of race/ethnicity on subsequent lung function. Preterm Non‐Hispanic Black and Hispanic White participants were compared with respect to neonatal factors and later lung function. Preterm Hispanic participants were born at a slightly lower gestational age, but in more recent birth years and with higher birth weight percentiles (Table 1). Hispanic preterm participants were also more likely to be treated with continuous positive pressure ventilation, diagnosed with BPD or sepsis, and required longer hospital length of stay (Table E1). Despite suggestion of a sicker Hispanic preterm population, significantly worse spirometric, volumetric, and diffusion capacity parameters were noted in the Non‐Hispanic Black preterm participants (Table 5). These differences persisted after adjusting for baseline differences (birth year, gestational age, weight for gestational age percentile as neonate, ever‐smoker, and BMI) using least squares multivariable regression. However, muscle forces and SGRQ respiratory symptoms were similar between groups after adjustment.

**Table 5:** Pulmonary function and respiratory symptoms by racial/ethnic background among preterm non‐hispanic black and preterm hispanic white participants.

Spirometry	Preterm Non‐Hispanic Black (*n* = 27)	Preterm Hispanic (*n* = 69)	Unadjusted *p*‐value a	Adjusted *p*‐value b
FEV1, *Z*‐Score	−0.97 (0.78)	0.56 (1.15)	**< 0.001**	**< 0.001**
FEV1, % predicted	86.4 (10.8)	107.2 (15.1)	**< 0.001**	**< 0.001**
FVC, Z‐Score	−0.69 (1.07)	1.29 (1.20)	**< 0.001**	**< 0.001**
FVC, % predicted	90.5 (15.0)	117.9 (16.6)	**< 0.001**	**< 0.001**
FEV1/FVC, *Z*‐score	−0.50 (1.12)	−1.06 (1.01)	**0.02**	0.93
FEV1/FVC ratio	81.4 (6.7)	80.1 (7.2)	0.39	0.68
FEF25‐75, *Z*‐score	−0.36 (0.86)	−0.60 (0.88)	0.23	0.98
FEF25‐75, % Predicted	91.0 (23.8)	87.7 (18.8)	0.47	0.80
**Lung volumes**				
TLC, *Z*‐score	−1.24 (0.95)	0.79 (1.21)	**< 0.001**	**< 0.001**
TLC, % Predicted	86.0 (10.8)	110.2 (16.1)	**< 0.001**	**< 0.001**
VC, *Z*‐score	−1.20 (1.20)	0.96 (1.75)	**< 0.001**	**< 0.001**
VC, % Predicted	85.6 (14.2)	111.4 (20.4)	**< 0.001**	**< 0.001**
IC, *Z*‐score	−0.47 (1.10)	1.30 (1.87)	**< 0.001**	**< 0.001**
IC, % Predicted	91.7 (19.8)	122.4 (33.0)	**< 0.001**	**< 0.001**
FRC, *Z*‐score	−1.11 (1.16)	−0.02 (1.19)	**< 0.001**	**< 0.001**
FRC, % Predicted	80.8 (20.3)	101.7 (24.8)	**< 0.001**	**< 0.001**
ERV, *Z*‐score	−0.91 (1.28)	0.13 (1.47)	**< 0.01**	0.17
ERV, % Predicted	75.4 (36.2)	107.9 (44.8)	**< 0.01**	0.13
RV, *Z*‐score	−0.45 (1.03)	0.13 (1.29)	**0.04**	**< 0.001**
RV, % Predicted	88.0 (30.9)	109.4 (50.8)	**0.04**	**< 0.001**
RV/TLC, *Z*‐score	0.08 (1.20)	−0.04 (1.31)	0.68	0.05
RV/TLC, % Predicted	103.0 (33.0)	98.8 (40.1)	0.63	0.05
**Diffusion capacity**				
DLCO, *Z*‐score	−1.55 (0.91)	−0.06 (0.89)	**< 0.001**	**< 0.001**
DLCO, % Predicted	81.0 (10.2)	99.8 (12.0)	**< 0.001**	**< 0.001**
DLCO Adj, *Z*‐score	−1.61 (0.93)	−0.18 (0.95)	**< 0.001**	**< 0.001**
DLCO Adj, % Predicted	80.3 (10.4)	98.1 (12.9)	**< 0.001**	**< 0.001**
VA, *Z*‐score	−2.09 (1.50)	0.30 (1.21)	**< 0.001**	**< 0.001**
VA, % Predicted	78.9 (14.4)	103.8 (13.2)	**< 0.001**	**< 0.001**
DLCO/VA, *Z*‐score	0.21 (1.22)	−0.44 (0.93)	**< 0.01**	**0.02**
DLCO/VA % Predicted	104.9 (16.9)	97.0 (12.1)	**0.01**	0.06
**Muscle forces**				
MIP, mmHg	71.0 (27.0)	70.9 (28.3)	0.99	0.28
MEP, mmHg	57.5 (24.1)	64.4 (28.8)	0.33	0.30
MVV, % Predicted	68.8 (20.0)	78.5 (20.7)	**0.04**	0.08
**St. George Respiratory Questionnaire**				
Symptom score (IQR)	14.5 (6.3–34.2)	6.8 (0–13.9)	**0.02**	0.17
Activity score (IQR)	30.4 (12.1–59.5)	12.3 (6.0–30.2)	**0.01**	0.07
Impact score (IQR)	7.5 (0–22.2)	1.6 (0–9.0)	**0.03**	0.47
Total score (IQR)	13.8 (6.7–35.9)	7.4 (2.5–15.9)	**< 0.01**	0.16

*Note:* Data are presented as mean (SD) unless otherwise specified. St. George respiratory questionnaire (SGRQ) data are presented as median (interquartile range). Percent predicted and Z‐scores are calculated using Global Lung Initiative (GLI) race neutral values, with exception of FEF25‐75 values for which GLI race neutral values were unavailable and GLI race‐specific datasets were used. Bold values are statistically significant at *p* < 0.05.

Abbreviations: DLCO = diffusion capacity for carbon monoxide, DLCO Adj = diffusion capacity for carbon monoxide adjusted for hemoglobin, ERV = expiratory reserve volume, FEV1 = forced expiratory volume in 1 s, FEF25‐75 = forced mid‐expiratory flow, FRC = functional residual capacity, FVC = forced vital capacity, IC = inspiratory capacity, MEP = maximal expiratory pressure, MIP = maximal inspiratory pressure, MVV = maximal voluntary ventilation, RV = residual volume, TLC = total lung capacity, VA = alveolar volume, VC = vital capacity.

^a^ Unadjusted P‐value calculated using unpaired t test (lung function variables) or calculated with Wilcoxon rank sum (SGRQ variables).

^b^ Adjusted P‐value calculated using least squares multivariable regression analysis adjusting for birth year, gestational age, weight for gestational age percentile as neonate, ever smoker(yes/no), and body mass index, as these were the variables different at baseline in the two populations.

### 3.4 Impact of Neonatal Factors

Next, we assessed whether neonatal factors were associated with subsequent lung function parameters (Table 6). BPD diagnosis was associated with lower FEV1 and higher RV/TLC, suggesting potential for an obstructive with air trapping phenotype. Low weight for gestational age percentile was associated with lower DLCO, consistent with known associations between intrauterine growth restriction and impaired pulmonary vascularization [17]. More recent birth years were associated with improved FVC but lower FEF25‐75 and FEV1/FVC with higher RV/TLC, suggesting potential for a dysanaptic phenotype. Black race was associated with worse FEV1, FVC, TLC, RV, RV/TLC, and DLCO, but not FEV1/FVC ratio, suggesting potential for a restrictive ventilatory phenotype.

**Table 6:** Preterm multivariable regression analysis of neonatal characteristics associated with pulmonary function measures.

Multivariable analysis variable	Beta (95% CI)	*p*‐value
**FEV‐1 *Z*‐score**		
Race (Hispanic)	0.33 (−0.41 to 1.08)	0.37
Race (Black)	−1.24 (−2.05 to −0.42)	**< 0.01**
Birth year	0.01 (−0.02 to 0.05)	0.42
Gestational age	0.01 (−0.08 to 0.10)	0.82
BPD	−0.64 (−1.26 to −0.02)	**0.04**
Weight for GA %ile	0.00 (−0.01 to 0.00)	0.45
**FVC Z‐score**		
Race (Hispanic)	0.32 (−0.47 to 1.10)	0.42
Race (Black)	−1.31 (−2.15 to −0.45)	**< 0.01**
Birth year	0.07 (0.03 to 0.11)	**< 0.01**
Gestational age	−0.02 (−0.12 to 0.07)	0.63
BPD	−0.62 (−1.27 to 0.03)	0.06
Weight for GA %ile	0.00 (−0.01 to 0.01)	0.61
**FEF25‐75 *Z*‐score**		
Race (Hispanic)	0.18 (−0.43 to 0.79)	0.56
Race (Black)	0.12 (−0.54 to 0.78)	0.71
Birth year	−0.03 (−0.06 to −0.01)	**0.02**
Gestational age	0.05 (−0.02 to 0.12)	0.17
BPD	−0.20 (−0.70 to 0.30)	0.43
Weight for GA %ile	0.00 (0.00 to 0.01)	0.74
**FEV‐1/FVC Z‐score**		
Race (Hispanic)	0.08 (−0.62 to 0.78)	0.82
Race (Black)	0.11 (−0.65 to 0.87)	0.77
Birth year	−0.07 (−0.11 to −0.04)	**< 0.01**
Gestational age	0.04 (−0.04 to 0.13)	0.31
BPD	−0.05 (−0.62 to 0.53)	0.88
Weight for GA %ile	0.00 (−0.01 to 0.01)	0.93
**TLC Z‐score**		
Race (Hispanic)	−0.42 (−1.27 to 0.43)	0.33
Race (Black)	−2.51 (−3.43 to −1.59)	**< 0.01**
Birth year	0.02 (−0.02 to 0.06)	0.34
Gestational age	0.02 (−0.08 to 012)	0.68
BPD	−0.19 (−0.89 to 0.51)	0.59
Weight for GA %ile	0.00 (−0.01 to 0.01)	0.64
**RV *Z*‐score**		
Race (Hispanic)	−0.93 (−1.81 to −0.05)	**0.04**
Race (Black)	−2.04 (−3.00 to −1.09)	**< 0.01**
Birth year	−0.08 (−0.12 to −0.03)	**< 0.01**
Gestational age	0.01 (−0.09 to 0.12)	0.80
BPD	0.57 (−0.15 to 1.30)	0.12
Weight for GA %ile	−0.01 (−0.01 to 0.00)	0.17
**RV/TLC Z‐score**		
Race (Hispanic)	−0.90 (−0.78 to −0.01)	**0.05**
Race (Black)	−1.34 (−2.31 to −0.38)	**< 0.01**
Birth year	−0.09 (−0.14 to −0.05)	**< 0.01**
Gestational age	0.00 (−0.11 to 0.10)	0.96
BPD	0.73 (−0.01 to 1.46)	**0.05**
Weight for GA %ile	−0.01 (−0.01 to 0.00)	0.20
**DLCO Adj *Z*‐Score**		
Race (Hispanic)	1.08 (0.46 to 1.70)	**< 0.01**
Race (Black)	−0.04 (−0.71 to 0.63)	0.90
Birth year	0.06 (0.03 to 0.09)	**< 0.01**
Gestational age	0.09 (0.01 to 0.16)	**0.03**
BPD	−0.36 (−0.87 to 0.16)	0.17
Weight for GA %ile	0.01 (0.00 to 0.01)	**0.02**

*Note:* Preterm individuals who were non‐Hispanic, nonblack were the reference value for the categorical variable of race in this model. *p*‐value calculated using least squares multivariable regression analysis adjusting for race, birth year, gestational age (GA), weight for gestational age percentile as neonate (Weight for GA %ile), and bronchopulmonary dysplasia (BPD). Models were not significant for respiratory forces measures, and thus data not shown. Bold values are statistically significant at *p* < 0.05.

### 3.5 Impact of Socioeconomic Factors

There is growing recognition of race as a social rather than biologic construct [18], and socioeconomic factors may influence lung development. Given the substantial racial/ethnic differences in our preterm population, and the availability of birth addresses in the NICU database, we evaluated all preterm participants in relation to childhood opportunity index (COI) scores defined by birth or adult geocoded addresses. Birth address COI *Z*‐scores were below national averages (*Z*‐score < 0) in both non‐Hispanic Black and Hispanic White preterm participants, consistent with the lower socioeconomic status of our county hospital, but were significantly lower in the preterm Black participants (Table E2). Thus, we assessed whether childhood deprivation was predictive of later pulmonary function independent of race/ethnicity, with multivariable regressions adjusted for birth year, gestational age, weight for gestational age percentile, ever smoker status, and BMI (Table E3). FEF25‐75 was inversely associated with COI variables (e.g. lowest COI score associated with better FEF25‐75), while RV, RV/TLC, and MEP were directly associated with COI score (e.g. lower COI score associated with lower RV/TLC). This suggests slightly better lung function among preterm participants with the most severe reduction in COI scores, and thus differences in lung function cannot be explained by differences in socioeconomic status captured by birth COI. No associations between pulmonary function and COI were identified when adult addresses were used for geocoding, consistent with the hypothesis that childhood exposures influence lung development and growth and thus late outcomes.

## 4 Discussion

We assessed long‐term pulmonary function in a unique U.S.‐born multi‐ethnic preterm cohort recruited utilizing the Parkland Hospital NICU Registry. Our primary objective was to evaluate whether adolescents and adults with a history of moderate to extreme prematurity have evidence of reduced lung function, as documented by comprehensive PFT including spirometry, lung volumes, diffusion capacity, and respiratory muscle forces. We identified that roughly one in three adults born preterm meet criteria for airflow obstruction or restriction, one in six have an abnormally low diffusion capacity, and one in four have impaired respiratory muscle strength. Further, spirometric, volumetric, and diffusion capacity measures were significantly lower in Black preterm participants as compared to Hispanic White preterm participants. Neonatal risk factors included history of BPD and lower weight for gestational age percentiles. Lung function differences between non‐Hispanic Black and Hispanic preterm participants were not well explained by neonatal differences or available socioeconomic factors evaluated by the birth address COI.

### 4.1 Recognition of Various Profiles After Preterm Birth

This study represents the first assessment of comprehensive PFT in a multi‐ethnic U.S. born preterm population, and to our knowledge the only assessment to include respiratory muscle force testing in addition to standard pulmonary function testing. Among the limited studies evaluating lung function in adults born moderately to extremely preterm, most were completed in cohorts born in the 1970s and 1980s in an era of neonatal care quite different from our cohort. These studies have reported airflow obstruction as the main lung function impairment after preterm birth which appears to persist across the early lifespan [1, 6, 19, 20, 21, 22, 23, 24]. For example, Vollsaeter et al. had two population‐based cohorts perform lung function testing and demonstrated airway obstruction that persisted from mid‐childhood to adulthood, particularly if there was history of neonatal BPD [23]. Doyle et al. reported lung function trajectories in an extremely preterm Australian cohort at eight, eighteen and 25 years of age, demonstrating that 38% of the preterm cohort had an FEV1/FVC ratio below the 5th percentile, higher than in our study (24%). Part of this witnessed difference may be attributed to our lower rate of extremely premature infants ( ~ 29%) [20] Meta‐analyses of preterm late adolescent and adult lung function also report decreased expiratory airflows indicating airflow obstruction, particularly in those with BPD, and report improvements over more recent birth years [1, 25, 26]. Doyle et al. reported an individual participant level meta‐analysis of spirometry for 935 preterm individuals aged 16–33 (mean 21 years) born with very low birth weight (≤ 1500 g) or very preterm (< 32 weeks) [6]. Mean Z‐scores for FEV1, FVC, and FEV1/FVC ratio were lower in the preterm group, with roughly 23% of preterm‐born adults demonstrating airflow obstruction with FEV1/FVC ratio below the lower limit of normal [6]. While restrictive ventilatory defects have not been highlighted in any of these studies and many did not include lung volume measures, the individual participant level meta‐analysis demonstrated 11% with FVC below the lower limit of normal [6], potentially suggesting restriction and similar to our PFT profile findings here. Several studies have also reported reduced diffusion capacity in adults born preterm, suggestive of reduced gas exchange [27, 28]. This may also suggest pulmonary vascular disease, and we have previously reported increased rates of mild pulmonary hypertension in adults born preterm [3]. Significant limitations of these prior studies include the absence of racial minorities (96.1% non‐Hispanic white in the individual participant meta‐analysis) and inclusion of primarily European and Australian datasets, as well as inclusion of studies primarily from the pre‐surfactant era with birth years before the early 1990s.

An additional novel finding in our study is the presence of significantly lower measures of respiratory muscle strength in the preterm population, which have not previously been described. A prior study demonstrated normal MVV %‐predicted in 70 preterm‐born female adolescents with average gestational age of 35 weeks [29]. However, only females were tested and the average gestational age is significantly older than that included in our study. Importantly, respiratory muscle forces including MIP and MEP correlate strongly with skeletal muscle strength as demonstrated by hand grip [30, 31], and indeed these were strongly correlated in our study as well. Lower handgrip and skeletal muscle strength have been reported in several studies of preterm physiology, and we have previously linked this to mitochondrial dysfunction in animal models of preterm birth [8, 32, 33, 34]. There are important implications to these novel findings. First, whether exercise training can improve measures of respiratory muscle weakness merits further evaluation. Two recent studies demonstrate improvement in peak oxygen consumption with exercise training in adults born preterm, but the direct effect on skeletal muscle strength was not reported [35, 36]. Second, clinicians should consider that individuals born preterm may be more likely to need bilevel rather than continuous positive airway pressure should they develop sleep disordered breathing or hypercarbia.

### 4.2 Impact of Race, Ethnicity and Social Factors

Very few studies address the impact of race on lung function after preterm birth, yet we identify strikingly lower lung function in Blacks born preterm. A recent study from the BPD Collaborative demonstrated higher rates of death or prolonged hospital stay in preterm infants with BPD born to Black mothers [37]. In the first year of life, two small studies have reported differences in airway resistance in preterm Black infants [38], while a third study reported reduced FEV0.5 and FVC with no difference in the ratio [39], potentially consistent with our report of higher rates of restrictive ventilatory patterns in Blacks born preterm. This particular pattern of restriction has likely been under recognized in the prematurity outcomes literature, possibly due to lack of inclusion of multi‐ethnic populations. However, mechanisms of ventilatory restrictive patterns are unclear. Prior analyses of the general population note that Black populations having lower sitting height, and sitting height accounts for 35%–53% of the racial differences in lung volumes [40, 41]. Whether preterm birth impacts sitting height or thoracic growth specifically is unclear, though its impact on height is well documented.

Prior studies have demonstrated an association between lower FEV1 and FVC values with markers of unfavorable environments and lower socioeconomic status [40, 41, 42, 43, 44]. However, how socioeconomic status intersects with postnatal lung function after preterm birth is unclear. We hypothesized that lower childhood COI, rather than adult COI, would be associated with worse lung function, as childhood exposures may influence lung growth. No associations between adult COI scoring and lung outcomes were noted potentially consistent with this hypothesis. An unexpected relationship between preterm birth COI was identified, with the lowest COI scores having better FEF25‐75, RV, and RV/TLC ratios, possibly indicating subtle improvement in airflow. The exact reasons for this relationship are unclear, and we suspect additional unmeasured factors contribute. We note that our study population represented the lowest quartiles nationally by COI scores, and did not include higher socioeconomic brackets due to the reference population of our county hospital. Further, those in the lowest brackets of socioeconomic status measures may have qualified for socialized aid programs which may have contributed to long term outcome differences. Regardless, racial differences in our preterm populations cannot be explained by environmental and social factors using the COI scoring and further research is needed. At this point, it is unclear how the transition to race‐neutral reference equations will impact diagnostic decisions in the preterm population who may have impaired lung growth over time. Additional attention to this is needed in future studies.

### 4.3 Assessment of Study Design and Methodology

Since the majority of pulmonary function outcome studies after moderate to extreme preterm birth come from international cohort studies, it is important to assess the generalizability of our study to the U.S. population. We propose that our inclusion of a multi‐ethnic preterm population born during a more modern era of neonatal care is a significant strength. Parkland Hospital is a county non‐referral hospital, and while staffed by academic neonatologists, the NICU does not accept higher level of care transfers, making the included population a reasonable sampling of real‐world data. The higher rates of cardiopulmonary morbidity and lower gestational age in the enrolled participants compared to the overall Parkland NICU database suggests our findings could over‐estimate the disease burden in the Parkland preterm population. However, we note BPD rates in the Parkland population have historically been low compared to national averages, as the Parkland NICU has participated in the Neonatal Research Network for several decades [45, 46]. Thus, it is also possible that our findings of most former preterm participants having normal lung function may over‐estimate health in a national sample with higher BPD rates. The availability of significant neonatal data is a major strength that permitted us to determine which neonatal risk factors were associated with pulmonary function outcomes, as the birth years of our cohort predate routine use of electronic health records which may be mined for more recent cohorts. Another strength is that we have interpreted PFT with respect to ventilatory patterns, rather than relying on single‐value differences between term and preterm individuals. A limitation of the study was poor matching by race/ethnicity in the term and preterm participants. Although we found that preterm Hispanic White individuals exhibited better lung function compared to preterm non‐Hispanic Blacks, we were unable to compare these former preterm populations to a term‐born group consisting of similar ethnic/racial background. However, given the movement away from inclusion of race/ethnicity in normative values, we do not believe this hinders the overall study conclusions.

## 5 Conclusion

In conclusion, we identified that roughly one in three adults born moderately to extremely preterm meet criteria for airflow obstruction or restriction, one in six have abnormally low diffusion capacity, and one in four have impaired respiratory muscle strength, with overall worse lung function in Black preterm participants as compared to Hispanic White preterm participants. Neonatal risk factors included history of BPD and lower weight for gestational age percentiles. Given that lung function differences between non‐Hispanic Black and Hispanic preterm participants were not well explained by neonatal differences or socioeconomic factors, future studies are needed to assess additional variables that may influence lung growth and subsequent function, including environmental exposures, nutrition and somatic growth.

## Subject Category

14.3 Neonatal Lung Disease & BPD.

## Author Contributions

**Nataly J. Sanchez‐Solano:** investigation, writing – original draft, methodology, writing – review and editing, formal analysis. **Tyler J. Wall:** investigation, writing – original draft, writing – review and editing, methodology, formal analysis. **Gregory P. Barton:** investigation, writing – original draft, methodology, writing – review and editing, conceptualization, formal analysis, project administration. **Dan M. Dane:** investigation, methodology, writing – review and editing. **Connie C. W. Hsia:** conceptualization, investigation, methodology, writing – review and editing. **Kara N. Goss:** conceptualization, investigation, funding acquisition, writing – original draft, methodology, validation, writing – review and editing, formal analysis, project administration, supervision, data curation.

## Ethics Statement

The study protocol was reviewed and approved by the UT Southwestern Institutional Review Board (STU 2020‐1332). All participants provided written consent, with assent obtained for adolescent participants.

## Conflicts of Interest

The authors declare no conflicts of interest.

## Supporting information


**Figure E1:** Correlation of respiratory muscle forces with handgrip strength. **Table E1:** Neonatal characteristics by racial/ethnic background among preterm non‐hispanic black and preterm hispanic white participants. **Table E2:** Childhood opportunity index z‐scores of preterm participants by race/ethnicity. **Table E3:** Preterm multivariable regression analysis of childhood opportunity index variable associated with pulmonary function measures.

## Data Availability

The data that support the findings of this study are available on request from the corresponding author. The data are not publicly available due to privacy or ethical restrictions.

## References

[1] S. J. Kotecha , J. T. D. Gibbons , C. W. Course , et al., “Geographical Differences and Temporal Improvements in Forced Expiratory Volume in 1 Second of Preterm‐Born Children: A Systematic Review and Meta‐Analysis,” JAMA Pediatrics 176 (2022): 867–877. 35759258 10.1001/jamapediatrics.2022.1990 PMC9237805

[2] D. S. Bui , J. L. Perret , E. H. Walters , et al., “Association Between Very to Moderate Preterm Births, Lung Function Deficits, and COPD at Age 53 Years: Analysis of a Prospective Cohort Study,” Lancet Respiratory medicine 10 (2022): 478–484. 35189074 10.1016/S2213-2600(21)00508-7

[3] K. N. Goss , A. G. Beshish , G. P. Barton , et al., “Early Pulmonary Vascular Disease in Young Adults Born Preterm,” American Journal of Respiratory and Critical Care Medicine 198 (2018): 1549–1558. 29944842 10.1164/rccm.201710-2016OC PMC6298636

[4] E. A. McGinn , E. W. Mandell , B. J. Smith , J. W. Duke , A. Bush , and S. H. Abman , “Dysanapsis as a Determinant of Lung Function in Development and Disease,” American Journal of Respiratory and Critical Care Medicine 208 (2023): 956–963. 37677135 10.1164/rccm.202306-1120PP PMC10870865

[5] B. Thébaud , K. N. Goss , M. Laughon , et al., “Bronchopulmonary Dysplasia,” Nature Reviews Disease Primers 5 (2019): 78. 10.1038/s41572-019-0127-7 PMC6986462 31727986

[6] L. W. Doyle , S. Andersson , A. Bush , et al., “Expiratory Airflow in Late Adolescence and Early Adulthood in Individuals Born Very Preterm or With Very Low Birthweight Compared With Controls Born at Term or With Normal Birthweight: A Meta‐Analysis of Individual Participant Data,” Lancet Respiratory medicine 7 (2019): 677–686. 31078498 10.1016/S2213-2600(18)30530-7

[7] N. J. Sanchez‐Solano , G. P. Barton , T. Martinez‐Fernandez , M. Lee , and K. N. Goss , “Sleep‐Disordered Breathing in a Multi‐Ethnic Cohort of Preterm Adolescents and Adults: Assessment of Neonatal and Subsequent Risk Factors,” Journal of Clinical Sleep Medicine 21 (2024): 519–528. 10.5664/jcsm.11440 PMC11874082 39492576

[8] G. P. Barton , A. Chandra , N. Sanchez‐Solano , J. D. Berry , and K. N. Goss , “Smaller Left Ventricular Size But Preserved Function in Adolescents and Adults Born Preterm,” Journal of the American Heart Association 13 (2024): e035529. 39248261 10.1161/JAHA.124.035529 PMC11935619

[9] P. W. Jones , F. H. Quirk , C. M. Baveystock , and P. Littlejohns , “A Self‐Complete Measure of Health Status for Chronic Airflow Limitation,” American Review of Respiratory Disease 145 (1992): 1321–1327. 1595997 10.1164/ajrccm/145.6.1321

[10] B. L. Graham , I. Steenbruggen , M. R. Miller , et al., “Standardization of Spirometry 2019 Update. An Official American Thoracic Society and European Respiratory Society Technical Statement,” American Journal of Respiratory and Critical Care Medicine 200 (2019): e70–e88. 31613151 10.1164/rccm.201908-1590ST PMC6794117

[11] S. Stanojevic , B. L. Graham , B. G. Cooper , et al., “Official ERS Technical Standards: Global Lung Function Initiative Reference Values for the Carbon Monoxide Transfer Factor for Caucasians,” European Respiratory Journal 50 (2017): 1700010. 28893868 10.1183/13993003.00010-2017

[12] S. Stanojevic , D. A. Kaminsky , M. R. Miller , et al., “ERS/ATS Technical Standard on Interpretive Strategies for Routine Lung Function Tests,” European Respiratory Journal 60 (2022): 2101499. 34949706 10.1183/13993003.01499-2021

[13] B. G. Cooper , J. Stocks , G. L. Hall , et al., “The Global Lung Function Initiative (GLI) Network: Bringing the World's Respiratory Reference Values Together,” Breathe 13 (2017): e56–e64. 28955406 10.1183/20734735.012717 PMC5607614

[14] D. Acevedo‐Garcia , C. Noelke , N. McArdle , et al., “Racial and Ethnic Inequities in Children′s Neighborhoods: Evidence From the New Child Opportunity Index 2.0,” Health Affairs 39 (2020): 1693–1701. 33017244 10.1377/hlthaff.2020.00735

[15] C. Noelke , N. McArdle , M. Baek , et al., Child Opportunity Index 2.0 Technical Documentation. 2020.

[16] J. R. Kaiser , J. M. Tilford , P. M. Simpson , W. A. Salhab , and C. R. Rosenfeld , “Hospital Survival of Very‐Low‐Birth‐Weight Neonates From 1977 to 2000,” Journal of Perinatology 24 (2004): 343–350. 15116138 10.1038/sj.jp.7211113

[17] P. J. Rozance , G. J. Seedorf , A. Brown , et al., “Intrauterine Growth Restriction Decreases Pulmonary Alveolar and Vessel Growth and Causes Pulmonary Artery Endothelial Cell Dysfunction in Vitro in Fetal Sheep,” American Journal of Physiology‐Lung Cellular and Molecular Physiology 301 (2011): L860–L871. 21873446 10.1152/ajplung.00197.2011 PMC3233832

[18] N. R. Bhakta , C. Bime , D. A. Kaminsky , et al., “Race and Ethnicity in Pulmonary Function Test Interpretation: An Official American Thoracic Society Statement,” American Journal of Respiratory and Critical Care Medicine 207 (2023): 978–995. 36973004 10.1164/rccm.202302-0310ST PMC10112445

[19] L. W. Doyle , A. M. Adams , C. Robertson , et al., “Increasing Airway Obstruction From 8 to 18 Years in Extremely Preterm/Low‐Birthweight Survivors Born in the Surfactant Era,” Thorax 72 (2017): 712–719. 27601432 10.1136/thoraxjnl-2016-208524

[20] L. W. Doyle , L. Irving , A. Haikerwal , K. Lee , S. Ranganathan , and J. Cheong , “Airway Obstruction in Young Adults Born Extremely Preterm or Extremely Low Birth Weight in the Postsurfactant Era,” Thorax 74 (2019): 1147–1153. 31558625 10.1136/thoraxjnl-2019-213757

[21] S. Caskey , A. Gough , S. Rowan , et al., “Structural and Functional Lung Impairment in Adult Survivors of Bronchopulmonary Dysplasia,” Annals of the American Thoracic Society 13 (2016): 1262–1270. 27222921 10.1513/AnnalsATS.201509-578OC

[22] M. Vollsæter , H. H. Clemm , E. Satrell , et al., “Adult Respiratory Outcomes of Extreme Preterm Birth. A Regional Cohort Study,” Annals of the American Thoracic Society 12 (2015): 313–322. 25616079 10.1513/AnnalsATS.201406-285OC

[23] M. Vollsæter , O. D. Røksund , G. E. Eide , T. Markestad , and T. Halvorsen , “Lung Function After Preterm Birth: Development From Mid‐Childhood to Adulthood,” Thorax 68 (2013): 767–776. 23749815 10.1136/thoraxjnl-2012-202980

[24] S. J. Kotecha , M. O. Edwards , W. J. Watkins , et al., “Effect of Preterm Birth on Later FEV1: A Systematic Review and Meta‐Analysis,” Thorax 68 (2013): 760–766. 23604458 10.1136/thoraxjnl-2012-203079

[25] J. T. D. Gibbons , C. W. Course , E. E. Evans , S. Kotecha , S. J. Kotecha , and S. J. Simpson , “Increasing Airway Obstruction Through Life Following Bronchopulmonary Dysplasia: A Meta‐Analysis,” ERJ Open Research 9 (2023): 00046‐2023. 37342090 10.1183/23120541.00046-2023 PMC10277871

[26] H. L. J. Lillebøe , M. S. Engeset , H. H. Clemm , et al., “Expiratory Airflow Limitation in Adults Born Extremely Preterm: A Systematic Review and Meta‐Analysis,” Paediatric Respiratory Reviews 50 (2024): 2–22. 38490917 10.1016/j.prrv.2024.02.002

[27] J. Yang , R. A. Kingsford , J. Horwood , et al., “Lung Function of Adults Born at Very Low Birth Weight,” Pediatrics 145 (2020): e20192359. 31900317 10.1542/peds.2019-2359

[28] P. Um‐Bergstrom , J. Hallberg , M. Pourbazargan , et al., “Pulmonary Outcomes in Adults With a History of Bronchopulmonary Dysplasia Differ From Patients With Asthma,” Respiratory Research 20 (2019): 102. 31126291 10.1186/s12931-019-1075-1 PMC6534852

[29] K. Kaczmarczyk , I. Wiszomirska , M. Szturmowicz , A. Magiera , and M. Błażkiewicz , “Are Preterm‐Born Survivors at Risk of Long‐Term Respiratory Disease?,” Therapeutic Advances in Respiratory Disease 11 (2017): 277–287. 28614994 10.1177/1753465817710595 PMC5933633

[30] K. Grigoriadis , I. Efstathiou , Z. Dimitriadis , et al., “Handgrip Force and Maximum Inspiratory and Expiratory Pressures in Critically Ill Patients With a Tracheostomy,” American Journal of Critical Care 30 (2021): e48–e53. 33644812 10.4037/ajcc2021248

[31] I. D. Efstathiou , I. P. Mavrou , and K. E. Grigoriadis , “Correlation Between Maximum Inspiratory Pressure and Hand‐Grip Force in Healthy Young and Middle‐Age Individuals,” Respiratory Care 61 (2016): 925–929. 27094394 10.4187/respcare.04319

[32] A. Deprez , R. El‐Jalbout , A. Cloutier , et al., “Adults Born Preterm Have Lower Peripheral Skeletal Muscle Area and Strength,” Scientific Reports 14 (2024): 21457. 39271745 10.1038/s41598-024-72533-6 PMC11399148

[33] K. M. Morrison , E. Gunn , S. Guay , J. Obeid , L. A. Schmidt , and S. Saigal , “Grip Strength Is Lower in Adults Born With Extremely Low Birth Weight Compared to Term‐Born Controls,” Pediatric Research 89 (2021): 996–1003. 32555537 10.1038/s41390-020-1012-5

[34] L. H. Tetri , G. M. Diffee , G. P. Barton , et al., “Sex‐Specific Skeletal Muscle Fatigability and Decreased Mitochondrial Oxidative Capacity in Adult Rats Exposed to Postnatal Hyperoxia,” Frontiers in Physiology 9 (2018): 326. 29651255 10.3389/fphys.2018.00326 PMC5884929

[35] B. Tardif , C. Mathieu , M. E. Caru , et al., “HAPI Fit: An Exercise Intervention to Improve Peak Aerobic Capacity in Young Adults Born Very Preterm,” Medicine and Science in Sports and Exercise 56 (2023): 44–52. 37707478 10.1249/MSS.0000000000003279 PMC11805472

[36] W. Williamson , A. J. Lewandowski , O. J. Huckstep , et al., “Effect of Moderate to High Intensity Aerobic Exercise on Blood Pressure in Young Adults: The TEPHRA Open, Two‐Arm, Parallel Superiority Randomized Clinical Trial,” eClinicalMedicine 48 (2022): 101445. 35706495 10.1016/j.eclinm.2022.101445 PMC9112102

[37] T. R. Lewis , M. J. Kielt , V. P. Walker , et al., “Association of Racial Disparities With In‐Hospital Outcomes in Severe Bronchopulmonary Dysplasia,” JAMA Pediatrics 176 (2022): 852–859. 35913704 10.1001/jamapediatrics.2022.2663 PMC9344383

[38] J. Stocks , M. Henschen , A.‐F. Hoo , K. Costeloe , and C. Dezateux , “Influence of Ethnicity and Gender on Airway Function in Preterm Infants,” American Journal of Respiratory and Critical Care Medicine 156 (1997): 1855–1862. 9412566 10.1164/ajrccm.156.6.9607056

[39] A. F. Hoo , A. Gupta , S. Lum , et al., “Impact of Ethnicity and Extreme Prematurity on Infant Pulmonary Function,” Pediatric Pulmonology 49 (2014): 679–687. 24123888 10.1002/ppul.22882 PMC4285893

[40] R. I. Harik‐Khan , J. L. Fleg , D. C. Muller , and R. A. Wise , “The Effect of Anthropometric and Socioeconomic Factors on the Racial Difference in Lung Function,” American Journal of Respiratory and Critical Care Medicine 164 (2001): 1647–1654. 11719304 10.1164/ajrccm.164.9.2106075

[41] R. I. Harik‐Khan , “Racial Difference in Lung Function in African‐American and White Children: Effect of Anthropometric, Socioeconomic, Nutritional, and Environmental Factors,” American Journal of Epidemiology 160 (2004): 893–900. 15496542 10.1093/aje/kwh297

[42] R. Holland , C. Bowerman , and S. Stanojevic , “The Contribution of Anthropometry and Socioeconomic Status to Racial Differences in Measures of Lung Function,” Chest 162 (2022): 635–646. 35469854 10.1016/j.chest.2022.04.017

[43] V. Rocha , S. Soares , S. Stringhini , and S. Fraga , “Socioeconomic Circumstances and Respiratory Function From Childhood to Early Adulthood: A Systematic Review and Meta‐Analysis,” BMJ Open 9 (2019): e027528. 10.1136/bmjopen-2018-027528 PMC6597002 31227536

[44] V. Rocha , S. Fraga , C. Moreira , et al., “Life‐Course Socioeconomic Disadvantage and Lung Function: A Multicohort Study of 70 496 Individuals,” European Respiratory Journal 57 (2021): 2001600. 33214206 10.1183/13993003.01600-2020

[45] N. Ambalavanan , M. Walsh , G. Bobashev , et al., “Intercenter Differences in Bronchopulmonary Dysplasia or Death Among Very Low Birth Weight Infants,” Pediatrics 127 (2011): e106–e116. 21149431 10.1542/peds.2010-0648 PMC3010091

[46] B. J. Stoll , N. I. Hansen , E. F. Bell , et al., “Neonatal Outcomes of Extremely Preterm Infants From the NICHD Neonatal Research Network,” Pediatrics 126 (2010): 443–456. 20732945 10.1542/peds.2009-2959 PMC2982806

