# Supplementary material for: Lung Function in a Multiethnic U.S. Cohort of Adolescents and Adults Born Preterm in the New BPD Era
Source: Pediatr Pulmonol. 2025 Aug 8;60(8):e71226. doi: 10.1002/ppul.71226 (PMC12333334; doi:10.1002/ppul.71226)
Supplement: Supplementary file 1 — Figure E1: Correlation of respiratory muscle forces with handgrip strength. Table E1: Neonatal characteristics by racial/ethnic background among preterm non‐hispanic black and preterm hispanic white participants. Table E2: Childhood opportunity index z‐scores of preterm participants by race/ethnicity. Table E3: Preterm multivariable regression analysis of childhood opportunity index variable associated with pulmonary function measures. [file PPUL-60-0-s001.docx]

**Online Supplement:**

Complete Methods:

*Participants*. The Cardiopulmonary Sequelae in Adolescents and Adults Born Preterm Study was a long-term follow up study from the Parkland Hospital Neonatal Intensive Care Unit (NICU) Registry. This single-center cross-sectional study prospectively enrolled term and preterm adolescents and young adults aged 12-40 from June 2021 to September of 2023. The complete study included health questionnaires, focused cardiopulmonary exam, echocardiography, comprehensive pulmonary function testing, and overnight oximetry; here we present results related to pulmonary function testing. Echocardiographic and sleep results have been previously published.(7, 8) The study protocol was reviewed and approved by the institutional review board. All participants provided a written consent, with assent obtained for adolescent participants.

Initiated in 1986, the Parkland NICU Registry contains extensive neonatal records for all infants requiring NICU care at Parkland Hospital, the primary safety-net hospital of Dallas County, Texas. Preterm participants were identified as eligible if born at ≤32 weeks gestation or with a birthweight of <1500 grams. Of the 6000 preterm subjects who were potentially eligible to participate, roughly 70% had available contact information and were mailed an invitation letter with instructions on how to schedule a study visit if interested. Neonatal data were obtained from the Parkland NICU Registry for preterm participants. Term participants were identified through a variety of measures, including a referral program where preterm participants could refer friends/family members; invitations through the University of Texas Southwestern electronic medical record; word of mouth; and local flyers. Term participants were eligible if they were born ≥37 weeks and had birthweight of >2500 g, with birth history self-reported. Term participants were excluded if they had a body mass index (BMI) >40 kg/m2, pre-existing cardiopulmonary disease, or use of cardiac or pulmonary medications in the prior 90 days.

*Data collection*. All participants attended a single study visit where data were collected to include demographics (age, gender, comorbidities), anthropometrics (height, weight, BMI, neck, and waist circumference), and vital signs. After familiarization with a handgrip testing device, handgrip testing was conducted in both hands to determine maximal voluntary contraction using Northstar echo grip (JTECH Medical, Midvale, UT). Participants completed the St. George Respiratory Questionnaire (SGRQ) to assess for respiratory symptomatology.(9) Fasting basic metabolic panel and hemoglobin were completed on a point of care testing device (iSTAT; Abbott Point of Care Inc, Abbott Park, IL), and blood banked for future use.

*Pulmonary function testing*. All participants completed the following comprehensive pulmonary function testing in accordance with ATS/ERS guidelines (10-12): (1) spirometry (FEV1= forced expiratory volume in 1 second; FVC= forced vital capacity; FEF25-75= forced mid-expiratory flow); (2) lung volumes (TLC= total lung capacity; VC= vital capacity; IC=inspiratory capacity; FRC= functional residual capacity; ERV= expiratory reserve volume; RV= residual volume); (3) lung diffusion capacity (DLCO= diffusion capacity for carbon monoxide; DLCO Adj= diffusion capacity for carbon monoxide adjusted for hemoglobin; VA= alveolar volume); and (4) respiratory forces (MIP= maximal inspiratory pressure; MEP= maximal expiratory pressure; MVV= maximal voluntary ventilation). Standard flow volume loops were completed on a Sensormedics (Yorba Linda, CA) 6200 Autobox Body Plethysmograph, following the ERS/ATS Standardization of Spirometry 2019 Update. Plethysmography was done on the same instrument. Immediately afterwards as part of the same session, single breath lung diffusing capacity was measured on a Sensormedics Vmax 229 Pulmonary Function/Cardiopulmonary Exercise testing instrument using the 2017 ERS/ATS standards, followed by measurement of MVV on the same instrument. Subjects were then coached to perform MIP and MEP maneuvers on a Marshalltown (Marshalltown, Iowa) pressure gauge. After one or two practice maneuvers, three trials of each were done and the highest value reached was reported. Predicted values were calculated using Global Lung Initiative (GLI) datasets.(13) GLI race neutral predicted and Z-score values are presented, with the exception of FEF25-75 values for which GLI race neutral values were unavailable and GLI race-specific datasets were used.

For disease classification, the ERS/ATS technical standard on interpretation was applied using Z-score defined upper and lower limits to define pathology following interpretation algorithms, noting the 5^th^ percentile as the lower limit of normal (LLN) and 95^th^ percentile as the upper limit of normal (ULN).(12) Specifically, obstructive pattern was defined by FEV1/FVC < LLN and FVC >LLN. Obstructive therefore included the dysanaptic pattern as well, which was defined as FEV1/FVC <LLN with FVC > ULN. Hyperinflation was defined as RV/TLC >ULN. Restriction was defined as TLC <LLN. Abnormally low DLCO was defined by DLCO <LLN, which was sub-classified into (1) primary vascular abnormality/emphysema, defined as DLCO <LLN with normal or elevated VA; (2) low lung volumes/poor expansion, defined as DLCO <LLN with VA <LLN and Kco >ULN; and (3) combined loss of capillary volume and lung volume, defined as DLCO <LLN with VA <LLN and Kco normal or low. Neuromuscular weakness was defined as MVV <70% predicted, plus MIP or MEP <60 mmHg.

For assessment of the impact of race on subsequent lung function outcomes, race was self-reported by all participants. Hispanic White and Non-Hispanic Black were the two primary ethnicity/race combinations in the preterm population, accounting for 91% of enrolled preterm participants. Thus, these two categories were compared, with adjustments made for differences in baseline characteristics between the preterm groups. In order to probe the contribution of socioeconomic factors, rather than race/ethnic background, on lung function, we geocoded all available addresses. Birth and current addresses were available for preterm-born participants, and only current addresses were available for term-born participants. Birth addresses were used to assess the birth environment and current address to assess the adult environment. Federal Information Processing Standard (FIPS) codes from the U.S. Census bureau website, which identify unique geographic areas, were obtained by individual address lookup via census.gov (https://geocoding.geo.census.gov/geocoder/geographies/address). Birth and current FIPS codes for each address were then matched to the Childhood Opportunity Index (COI) 2.0 2015 dataset, retrieved December 6, 2023.(14, 15) The COI 2.0 is a composite index measured at the census tract level to capture neighborhood resources and conditions identified to be important for child health and development. Nationally standardized Z-scores for subdomains of education, health and environment, social and economic as well as a total COI score was computed for all participants. Because no associations with COI scores could be identified in term participants, only preterm participant data are shown.

*Statistical analysis.* Statistical analysis was performed using Graphpad Prism version 10 (GraphPad Software Inc., La Jolla, CA, USA). A total of 153 participants were included. Univariable comparisons of baseline/demographic characteristics between term and preterm group as well as preterm non-Hispanic Black and Hispanic groups was conducted using Chi-square test for categorical variables, and unpaired *t*-test (or its non-parametric counterpart, Wilcoxon rank sum test if necessary) for continuous variables. Term and preterm pulmonary function and respiratory symptoms comparisons were constructed in a similar fashion. Odds ratios were computed using Baptist-Pike method for various pulmonary function profiles based on birth history grouping. Subgroup analysis was conducted within preterm group (n=96) to evaluate associations of various neonatal factors and socioeconomic factors with long-term lung function. One preterm participant was withheld from the subgroup analysis given endorsement of both Black race and Hispanic ethnicity. Univariable comparisons of pulmonary function tests, respiratory symptoms and average COI scores between preterm non-Hispanic Black and Hispanic populations were conducted using unpaired t-test (or Mann-Whitney test if necessary) for continuous variables. Least squares multivariable regression analyses were completed between the preterm groups to assess for the impact of neonatal and socioeconomic factors. Multicolinearity was assessed using the Variance Inflation Factor(VIF) with all values being less than 2.5.

**Figure E1. Correlation of respiratory muscle forces with handgrip strength.** MIP = maximal inspiratory pressure. MEP = maximal expiratory pressure. MVV = maximal voluntary ventilation.

**Table E1. Neonatal Characteristics By Racial/Ethnic Background Among Preterm Non-Hispanic Black and Preterm Hispanic White Participants**

| **Characteristic** | **Preterm Non-Hispanic Black**  **(N=27)** | **Preterm Hispanic**  **(N=69)** | **P Value** |
| --- | --- | --- | --- |
| Neonatal diagnoses |  |  |  |
| *Respiratory* |  |  |  |
| IMV, median (IQR), days | 1 (4) | 1 (9) | 0.47 |
| NIV, median (IQR), days | 0 (3) | 2 (16) | **0.02** |
| Total Ventilation, median (IQR), days | 2(5) | 4 (25) | 0.15 |
| Duration of Oxygen Therapy, median (IQR), days | 3 (15) | 5 (48) | 0.58 |
| Bronchopulmonary Dysplasia, *n* (%) | 1 (4) | 15 (22) | **0.04** |
| Antenatal Steroids, n (%)* | 2 (29) | 31 (61) | 0.22 |
| Surfactant, n (%)* | 2 (20) | 27 (49) | 0.16 |
| Chronic Lung Disease by CXR, *n* (%) | 5 (19) | 23 (33) | 0.21 |
| Apnea of Prematurity, *n* (%) | 16 (59) | 47 (68) | 0.48 |
| *Cardiovascular* |  |  |  |
| Patent Ductus Arteriosus, *n* (%) | 5 (19) | 17 (25) | 0.60 |
| Congenital Heart Disease, *n* (%) | 1 (4) | 4 (6) | 1.0 |
| *Neurological* |  |  |  |
| Intraventricular Hemorrhage, *n* (%) |  |  |  |
| No Intraventricular Hemorrhage | 18 (67) | 54 (78) | 0.30 |
| Grade I-II | 5 (19) | 12 (17) | 1.0 |
| Grade III-IV | 4 (15) | 3 (4) | 0.09 |
| *Infectious* |  |  |  |
| Sepsis, *n* (%) | 6 (22) | 34 (49) | **0.02** |
| Necrotizing Enterocolitis, n (%) | 2 (7) | 6 (9) | 1.0 |
| Total Length of Stay, median (IQR), days | 31 (37) | 60 (57) | **0.04** |

- Data are presented as n (%) or mean (SD) unless otherwise specified.

- Definition of abbreviations: IMV= invasive mechanical ventilation; IQR= interquartile ranges; NIV= noninvasive mechanical ventilation; CXR=chest x-ray

- P-value calculated comparing preterm vs. term with Wilcoxon rank sum test for non-normally distributed continuous variables, T-test for normally distributed continuous variables, and chi-squared for categorical variables.

*There were 46 and 39 missing data points for antenatal steroids and surfactant, respectively.

**Table E2. Childhood Opportunity Index Z-scores of Preterm Participants by Race/Ethnicity**

| **Childhood Opportunity Index (COI) 2015** | **Preterm Non-Hispanic Black**  **(n=27)** | **Preterm Hispanic**  **(n=69)** | **P-Value** |
| --- | --- | --- | --- |
| Education Z-Score | -0.04 (0.04) | -0.03 (0.03) | 0.06 |
| Health/Environment Z-Score | -0.06 (0.04) | -0.04 (0.03) | **0.03** |
| Social/Economic Z-score | -0.23 (0.18) | -0.09 (0.15) | **<0.01** |
| COI Total Z-score | -0.04 (0.03) | -0.02 (0.02) | **<0.01** |

- Data are presented as mean (SD). COI = childhood opportunity index.

- P-value calculated using unpaired t test, unadjusted.

**Table E3. Preterm Multivariable Regression Analysis of Childhood Opportunity Index Variable Associated with Pulmonary Function Measures**

| **Multivariable Analysis Variable** | **Beta (95% CI)** | **P-Value** |
| --- | --- | --- |
| **FEV-1 Z-score** |  |  |
| Education Z-score | -3.07 (-10.16 – 4.02) | 0.39 |
| Health/Environment Z-score | -6.50 (-12.99 – -0.003) | **0.049** |
| Social/Economic Z-score | -0.78 (-2.25 – 0.69) | 0.29 |
| COI Total Z-score | -6.41 (-16.31 – 3.50) | 0.20 |
| **FVC Z-score** |  |  |
| Education Z-score | 0.28 (-7.23 – 7.79) | 0.94 |
| Health/Environment Z-score | -3.77 (-10.72 – 3.18) | 0.28 |
| Social/Economic Z-score | -0.39 (-1.95 – 1.16) | 0.61 |
| COI Total Z-score | -2.96 (-13.49 – 7.57) | 0.58 |
| **FEF25-75 Z-score** |  |  |
| Education Z-score | -3.96 (-8.87 – 0.94) | 0.11 |
| Health/Environment Z-score | -5.02 (-9.54 – -0.50) | **0.03** |
| Social/Economic Z-score | -1.16 (-2.17 – -0.16) | **0.02** |
| COI Total Z-score | -8.29 (-15.07 – -1.51) | **0.02** |
| **FEV-1/FVC Z-score** |  |  |
| Education Z-score | -4.56 (-10.20 – 1.08) | 0.11 |
| Health/Environment Z-score | -3.10 (-8.38 – 2.19) | 0.25 |
| Social/Economic Z-score | -0.64 (-1.82 – 0.54) | 0.29 |
| COI Total Z-score | -5.16 (-13.11 – 2.80) | 0.20 |
| **TLC Z-score** |  |  |
| Education Z-score | 4.03 (-4.18 – 12.24) | 0.33 |
| Health/Environment Z-score | 3.12 (-4.77 – 11.02) | 0.43 |
| Social/Economic Z-score | 1.29 (-0.40 – 2.99) | 0.13 |
| COI Total Z-score | 8.57 (-2.96 – 20.10) | 0.14 |
| **RV Z-score** |  |  |
| Education Z-score | 4.55 (-3.44 – 12.54) | 0.26 |
| Health/Environment Z-score | 7.90 (0.35 – 15.45) | **0.04** |
| Social/Economic Z-score | 1.98 (0.36 – 3.60) | **0.02** |
| COI Total Z-score | 13.50 (2.46 – 24.53) | **0.02** |
| **RV/TLC Z-score** |  |  |
| Education Z-score | 4.19 (-3.39 – 11.76) | 0.28 |
| Health/Environment Z-score | 8.17 (1.05 – 15.29) | **0.03** |
| Social/Economic Z-score | 1.82 (0.28 – 3.36) | **0.02** |
| COI Total Z-score | 12.60 (2.14 – 23.06) | **0.02** |
| **DLCO Adj Z-Score** |  |  |
| Education Z-score | 3.65 (-2.13 – 9.43) | 0.21 |
| Health/Environment Z-score | -2.88 (-8.30 – 2.55) | 0.30 |
| Social/Economic Z-score | -0.14 (-1.36 – 1.07) | 0.82 |
| COI Total Z-score | -0.65 (-8.90 – 7.60) | 0.88 |
| **MIP Score** |  |  |
| Education Z-score | 150.0 (-9.65 – 309.7) | 0.07 |
| Health/Environment Z-score | 124.8 (-26.61 – 276.1) | 0.11 |
| Social/Economic Z-score | 22.40 (-11.16 – 55.97) | 0.19 |
| COI Total Z-score | 182.0 (-44.07 – 408.0) | 0.11 |
| **MEP Score** |  |  |
| Education Z-score | 194.7 (23.04 – 366.4) | **0.03** |
| Health/Environment Z-score | 108.0 (-57.08 – 273.1) | 0.20 |
| Social/Economic Z-score | 43.0 (7.40 – 78.60) | **0.02** |
| COI Total Z-score | 297.8 (57.32 – 538.2) | **0.02** |
| **MVV % Predicted** |  |  |
| Education Z-score | 14.08 (-108.0 – 136.1) | 0.82 |
| Health/Environment Z-score | -43.86 (-157.6 – 69.87) | 0.45 |
| Social/Economic Z-score | -4.62 (-30.0– 20.75) | 0.72 |
| COI Total Z-score | -32.21 (-204.1 – 139.7) | 0.71 |
| **SGRQ Symptom Score** |  |  |
| Education Z-score | -90.03 (-189.9 – 9.87) | 0.08 |
| Health/Environment Z-score | 58.29 (-35.84 – 152.4) | 0.22 |
| Social/Economic Z-score | 0.37 (-20.74 – 21.49) | 0.97 |
| COI Total Z-score | -3.75 (-146.8 – 139.3) | 0.96 |
| **SGRQ Activity Score** |  |  |
| Education Z-score | -34.32 (-160.4 – 91.79) | 0.59 |
| Health/Environment Z-score | -16.34 (-134.3 – 101.6) | 0.78 |
| Social/Economic Z-score | 2.12 (1.47 – 2.77) | **<0.01** |
| COI Total Z-score | -64.65 (-242.1 – 112.8) | 0.47 |
| **SGRQ Impact Score** |  |  |
| Education Z-score | -64.74 (-134.3 – 4.79) | 0.07 |
| Health/Environment Z-score | 15.52 (-50.49 – 81.53) | 0.64 |
| Social/Economic Z-score | -2.16 (-16.86 – 12.55) | 0.77 |
| COI Total Z-score | -22.05 (-121.6 – 77.51) | 0.66 |
| **SGRQ Total Score** |  |  |
| Education Z-score | -58.39 (-137.5 – 20.73) | 0.15 |
| Health/Environment Z-score | 11.41 (-63.28 – 86.11) | 0.76 |
| Social/Economic Z-score | -4.00 (-20.62 – 12.61) | 0.63 |
| COI Total Z-score | -31.51 (-144.0 – 81.0) | 0.58 |

- Data are presented as beta coefficient with 95% confidence interval. P-value calculated using least squares multivariable regression analysis adjusting for birth year, gestational age, weight for gestational age percentile as neonate, ever smoker(yes/no), and body mass index, as these were the variables different at baseline in the two populations.

- Definition of abbreviations: FEV1= forced expiratory volume in 1 second; FVC= forced vital capacity; FEF25-75= forced mid-expiratory flow; TLC= total lung capacity; VC= vital capacity; IC=inspiratory capacity; FRC= functional residual capacity; ERV= expiratory reserve volume; RV= residual volume; DLCO= diffusion capacity for carbon monoxide; DLCO Adj= diffusion capacity for carbon monoxide adjusted for hemoglobin; VA= alveolar volume; MIP= maximal inspiratory pressure; MEP= maximal expiratory pressure; MVV= maximal voluntary ventilation.
